# Technegas, A Universal Technique for Lung Imaging in Nuclear Medicine: Technology, Physicochemical Properties, and Clinical Applications

**DOI:** 10.3390/pharmaceutics15041108

**Published:** 2023-03-30

**Authors:** Isra Khatib, Paul M. Young

**Affiliations:** 1 Ab Initio Pharma Pty Ltd., 67-73 Missenden Road, Camperdown, NSW 2050, Australia; esraa.al-khatib@ab-initio-pharma.com; 2 Woolcock Institute of Medical Research, 431 Glebe Point Road, Glebe, NSW 2037, Australia; 3 Macquarie Business School, Macquarie University, NSW 2109, Australia

**Keywords:** argon, lung imaging/ventilation, technetium-99m-pertechnetate, particle size, Pertechnegas, pulmonary embolism, radioactive aerosol, Technegas particles

## Abstract

Technegas was developed in Australia as an imaging radioaerosol in the late 1980s and is now commercialized by Cyclomedica, Pty Ltd. for diagnosing pulmonary embolism (PE). Technegas is produced by heating technetium-99m in a carbon crucible for a few seconds at high temperatures (2750 °C) to generate technetium–carbon nanoparticles with a gas-like behaviour. The submicron particulates formed allow easy diffusion to the lung periphery when inhaled. Technegas has been used for diagnosis in over 4.4 m patients across 60 countries and now offers exciting opportunities in areas outside of PE, including asthma and chronic obstructive pulmonary disease (COPD). The Technegas generation process and the physicochemical attributes of the aerosol have been studied over the past 30 years in parallel with the advancement in different analytical methodologies. Thus, it is now well established that the Technegas aerosol has a radioactivity aerodynamic diameter of <500 nm and is composed of agglomerated nanoparticles. With a plethora of literature studying different aspects of Technegas, this review focuses on a historical evaluation of the different methodologies’ findings over the years that provides insight into a scientific consensus of this technology. Also, we briefly discuss recent clinical innovations using Technegas and a brief history of Technegas patents.

## 1. Introduction

**Figure 1:**
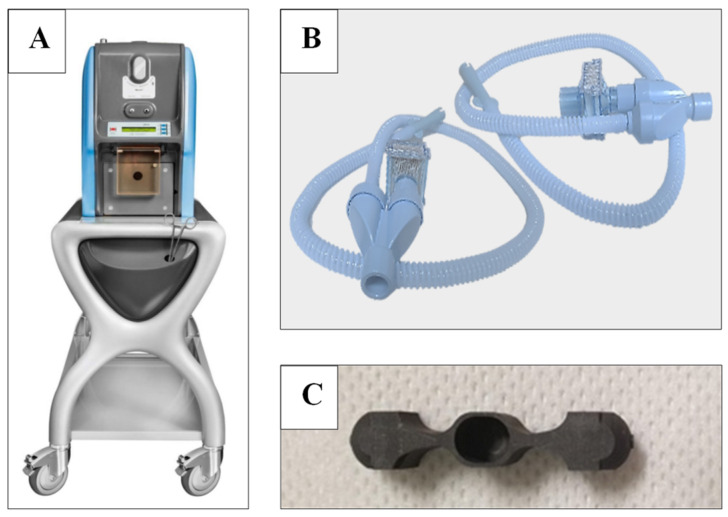
Photograph of (**A**) TechnegasPlus Technegas^®^ generator, (**B**) Cyclopharm Patient Administration Set (PAS), and (**C**) Pulmotec^®^ empty crucible. (Courtesy of Cyclomedica.)

In 1984, Technegas was developed in Australia as a respiratory imaging and diagnostic tool. Technegas is an ultrafine nanoparticle dispersion of technetium-labelled carbon, produced by heating technetium-99m in a carbon crucible for a few seconds at high temperatures using a Technegas generator (Figure 1A). Due to their small size and hydrophobic properties, generated Technegas nanoparticles have a gas-like behaviour, allowing diffusion to the periphery of the lung when inhaled. The resulting alveolar penetration allows unprecedented scintigraphy imaging for the diagnosis of pulmonary embolism and many other respiratory disorders. Historically, prior to commercialisation in 1989, Technegas was referred to as “Pseudogas”. The term Pseudogas was used since the ultrafine monodisperse aerosol particles generated by high-temperature combustion of Tc^99m^ diffuses in a gas-like manner in the lung and thus behaved more like a molecular gas than conventional inhaled aerosol [1,2]. Initial clinical studies using Pseudogas revealed that patients could inhale the necessary activity in one or two breaths, and even severely ill patients could accomplish dosing through passive breathing in less than a minute. In addition, the gas-like nature of the technetium particulates ensured minimal bronchial deposition in all but severe COPD patients.

**Figure 2:**
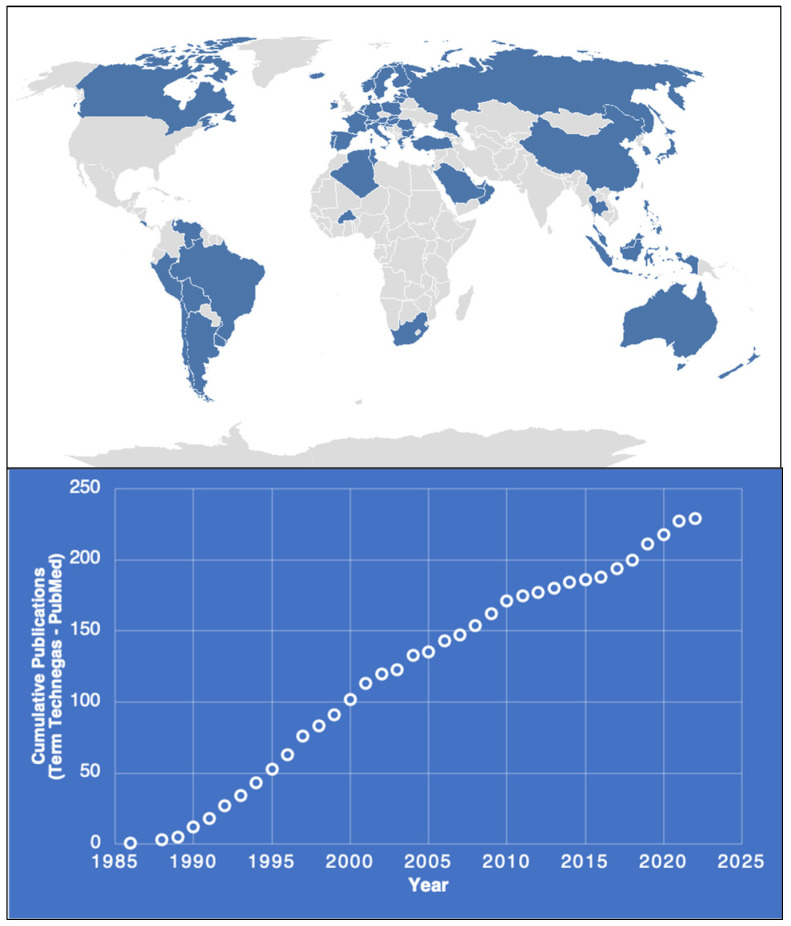
Global registration of Technegas as of August 2022 (blue areas on the map) and cumulative publication record as a function of year (PubMed Technegas).

By 1986, the term Technegas was already being used clinically in Australia to describe the Pseudogas agent. Technegas rapidly became the preference for diagnostic ventilation imaging. As of 2022, Technegas has been registered in more than 60 countries worldwide (Figure 2). As of June 2022, Cyclopharm has 1437 devices actively in use globally with testing completed in over 4.4 m patients. Furthermore, there are over 230 publications relating to Technegas as identified by PubMed in 2022 and over 2400 wider publications including abstracts, media posts, and patents as reported by Google Scholar.

The continued research within the field as highlighted by Figure 2 shows that the technology is not only current but embraced as a tool by the clinical community globally. More recently, with the recent COVID-19 pandemic, there have been significant issues raised with clinical personnel conducting treatment or diagnostics that involve forced respiratory manoeuvres, inhalation of nebulized products, or procedures that risk aspiration of the virus. Interestingly, the general consensus for Technegas has been favourable in this environment since the aerosol behaves as a gas rather than a particulate system (such as used with conventional aerosol generators), and because only two or three puffs are necessary to get adequate images, this system greatly reduces potential COVID-19 exposure to technologists [3]. Furthermore, while the primary function of Technegas has been in the diagnosis of pulmonary embolism, the technology has been applied to a number of exciting applications, including use in chronic thromboembolic pulmonary hypertension, asthma, chronic obstructive pulmonary disease, and emphysema [4].

With over 35 years of clinical application and global use, it is interesting to note that the United States of America remains one of the remaining jurisdictions in the Western world where Technegas is not registered. This is despite calls to the Federal Drug Administration (FDA) from the clinical community to fast-track approval of this technology [5]. Cyclopharm submitted a new drug application (NDA) for Technegas in 2020 and is currently addressing a complete response letter (CRL) that raised issues related to the unique characteristics, production, and delivery of Technegas [6]. With decades of clinical use and scientific evaluation, we present a historical review of the Technegas technology, with a focus on the underlying principles of the technology and the physical/chemical attributes of the Technegas aerosol in terms of its formation, dosimetry, size, and composition. We also address Technegas clinical adoption and applications globally.

## 2. Technology

### 2.1. Principles of Operation

In general, the operation of Technegas for clinical use in lung imaging is composed of three stages: (1) the simmer, (2) the burn, and (3) inhalation by the patient. The simmer stage involves evaporating the liquid carrier that the radiotracer is presented in using a graphite crucible. The burn stage uses resistive heating under a pure argon atmosphere to generate the Technegas aerosol. In the inhalation stage, Technegas particles are inhaled and deposited in the sub-segmental areas of the lung and trapped by a surfactant in the alveolar walls below the conducting airways [7].

Technegas operation requires preparatory steps and an understanding of the operation principles from the technicians as well as the patients. During the production of Technegas, the generator is attached to a supply of argon and is mains powered (for heating the crucible to high temperature). However, after gas production, the generator is powered by a battery and can be transferred to the patient’s bedside for the delivery of the gas. This grants better protection for the patients, staff, and imaging area from radiation since the gas is produced remotely. The commercial radiation source for operating Technegas generators is ^99m^Tc-sodium pertechnetate solution prepared in saline. Production of the Technegas is conducted by heating the ^99m^Tc-sodium pertechnetate in a graphite crucible (Figure 1C) to high temperatures [8].

**Figure 3:**
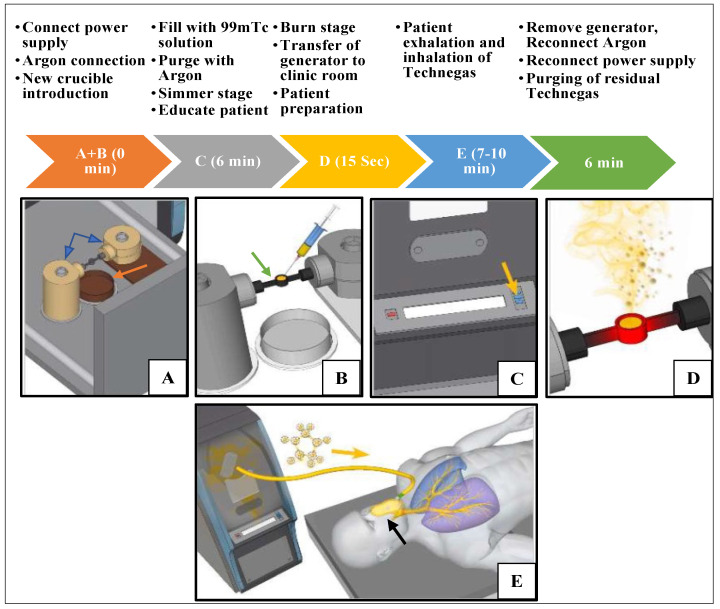
Principles for the operation of Technegas generator for lung imaging. (**A**) Contacts (blue arrows) and collection tray (orange arrow), (**B**) Pulmotec^®^ graphite crucible (green arrow), (**C**) start simmering button (yellow arrow), (**D**) burn stage, (**E**) inhalation stage using patient administration set (black arrow). (Inset courtesy of Cyclomedica.)

A simplified overview of the clinical operating steps is outlined in Figure 3. Briefly, a Pulmotec™ graphite crucible is inserted into the generator, and the system is switched on and connected to an argon source. Prior to use, new crucible surfaces are wetted with ethanol to aid evaporation of the added ^99m^Tc solution, reduce the risk of displacement from the crucible well upon heating, and displace any moisture that may reside on the crucible surface, especially any contributing oxygen (Figure 3A). Using a syringe (Figure 3B), a volume between 0.10 and 0.14 mL ^99m^Tc sodium pertechnetate solution with radioactivity in the range of 250–900 MBq is added. To avoid producing a free pertechnetate that may deteriorate imaging quality, the liquid meniscus should be either flat or concave within the well (to avoid the ^99m^Tc being ‘puffed off’ from the surface of the crucible, leading to a wet aerosol of free pertechnetate). In an ultrapure argon environment, the liquid ^99m^Tc is evaporated at 70 °C for 6 min (this is referred to as the simmer stage) (Figure 3C) so that a white crust of salt and pertechnetate is formed on the graphite surface. To generate the Technegas, a burn stage (Figure 3D) is initiated after simmer completion. The process uses an oscillating current arc formed between the terminals carrying the crucible to ablate both graphite and ^99m^Tc. Meanwhile, the temperature is increased to 2750 °C within 2 s and maintained for 15 s in an ultrapure argon environment to combine ^99m^Tc and carbon into nanoparticles. The yield of Technegas after this process is greater than 40% of the supplied ^99m^Tc (UM.US-7.EN). During the burn stage, the generator is connected to the mains power supply. Once the burn stage is complete, the generator can be unplugged from the mains and transferred to imaging for the ventilation or inhalation stage (Figure 3E). The ventilation/inhalation process occurs using a patient administration set (PAS) (Figure 1B) and must be initiated after the burn stage within a 10 min window. The inhalation process length and frequency depend on the patient reaching a recommended dose of approximately 1500 to 2500 counts per second. This inhalation window was suggested as a prolonged storage of Technegas may result in agglomeration of Technegas into larger particles along with losses on the walls of the Technegas generator chamber. The patient is educated during the initial preparatory process so that they coordinate their inhalation with release of the Technegas, with a breath hold and release after taking each dose. After completing the inspiration stage, argon is reconnected, and the generator plugged back into the main power supply for a 6 min purging cycle. This allows residual Technegas in the chamber to be cleared through the system filter, thus making the generator ready for the next operation [8].

**Figure 4:**
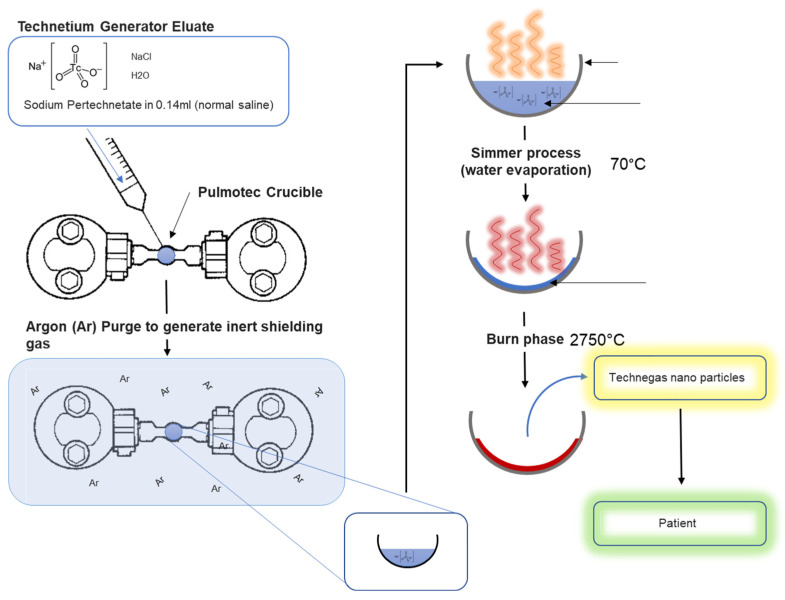
Macroscopic process of Technegas generation process at the crucible level.

A schematic of the process of Technegas generation at the macroscopic crucible level is shown in Figure 4.

### 2.2. Production of Technegas Theory

**Figure 5:**
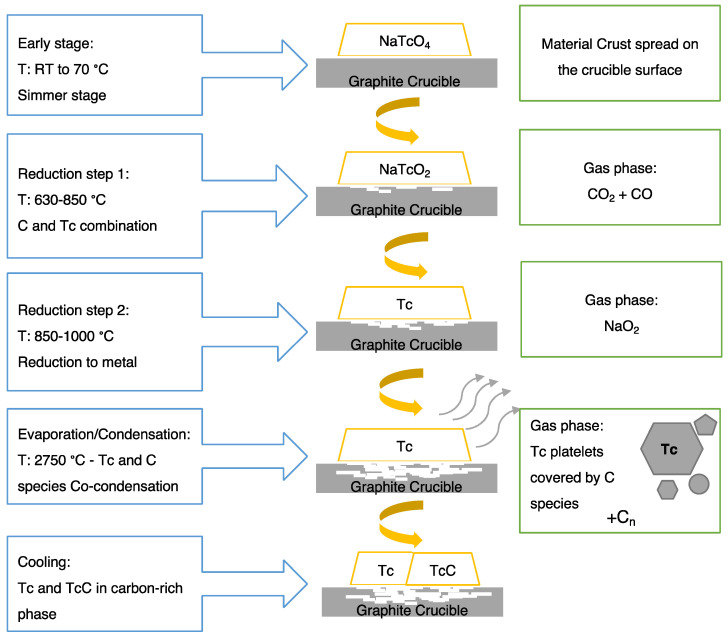
A schematic illustration represents the proposed Technegas production mechanism. Modified from the original study of potassium pertechnetate. Reprinted/adapted from Ref. [7].

The macroscopic process of Technegas production at a crucible level is shown in Figure 4. At a molecular level, a mechanism for Technegas generation was proposed by Senden et al. [7]. In this landmark paper, Senden et al. evaluated the physicochemical properties of potassium pertechnetate (K^99^TcO_4_), when heated in a graphite crucible, using thermal gravimetric analysis, X-ray diffraction, transmission electron, and atomic force microscopy. While sodium pertechnetate (Na^99^TcO_4_) is currently used in the TechnegasPlus generator, the chemistry with the potassium salt will be similar since the formation of Technegas particles occurs after reduction of the pertechnetate to the metal. A schematic of the Na^99^TcO_4_ reduction process during rapid heating is shown in Figure 5.

Using thermogravimetric analysis, combined with X-ray crystallography and microscopy, Senden et al. proposed the following mechanism for the formation of Technegas (modified here to incorporate the sodium salt). Initially, ^99m^Tc sodium pertechnetate is present as an aqueous solution in sodium chloride (saline). During the simmer stage (70 °C), water evaporates to leave a residual precipitate of ^99m^Tc sodium pertechnetate along with sodium chloride from the saline. This radioactive material is presented as a white crust on the surface of the graphite crucible. The crucible was then heated rapidly to ≥ 2250 °C [8] (today, all Technegas generators are calibrated to heat to 2750 °C). During this process of rapid heating, the following proposed chemistry occurs:

At 630–850 °C, the carbon (C) substrate combines with pertechnetate (TcO_4_) resulting in the release of carbon dioxide (CO_2_) and carbon monoxide (CO), which is followed by a second reduction to technetium metal at 850–1000 °C with the release of sodium dioxide. As the temperature continues to rise to 2250 °C, Tc platelets are formed, and their surfaces are covered with various C species via co-condensation, which is then liberated as a gas phase containing Cn species and carbon-rich Tc particles [7]. 

The overall reduction of the Na^99^TcO_4_ can be formulated in the following equation:

2NaTcO_4_ + 3C → CO_2_ ↑ + 2CO ↑ + 2NaTcO_2_ → 2NaO_2_ ↑ + 2Tc

Senden et al. observed that the encapsulation process of Tc with C occurs after the crystallization of the metallic aerosol. At 2100 °C, the released activity from the crucible is 20%; however, this rises rapidly above 2350 °C, with more than 80% production at temperatures above 2550 °C. The retained activity in the burnt crucible was shown to be around 25% with around half of the produced material being available for inhalation (with the remainder being collected on the walls of the chamber during the 3 min cooling-down stage post-burn) [7]. Today, Technegas generators operate to a heating temperature of 2750 °C to ensure optimum production efficiency. Fawdry and Gruenewald found that 86 ± 4% of the loaded activity in the graphite furnace was produced as Technegas and the amount available for inhalation by the patients was 70% of the loaded activity. The losses to the chamber were reported as being less than 30% after 10 min of generation. Subsequently, as long as patients are easily ventilated within minutes of gas generation, chamber losses are not a problem [9]. The overall chemistry occurs rapidly with the burn process, and in current commercial systems the temperature rises to 2750 °C within 2 s with the whole burn occurring for 15 s.

### 2.3. Patents—Device and Particles

Patents disclosed relating to the production method and apparatus of Technegas as an inhalable radionuclide labelled aerosol are:

#### 2.3.1. Method of Forming a Radioactive Metallic Vapor—US 5064634

The patent discloses the invention of a method for producing an inhalable diagnostic aerosol for the purpose of diagnosing respiratory dysfunction by loading a pharmaceutical radionuclide onto a carbon crucible followed by exposing the crucible to resistive heating. The patent also described the apparatus applied in the production of the diagnostic aerosol of the invention. In this patent, the heating of the carbon crucible with radioactive material under inert gas atmosphere was at temperatures in the region of at least 1900 ° C and, more preferably, to at least 2200 °C to produce an inhalable composition [10]. 

#### 2.3.2. Device for Producing a Gas-Lite Radionuclide Composition—US 5228444

The patent discloses the production method and apparatus of such radionuclides with a carbon crucible heated to a temperature within the range of 1500 °C to 2500 °C. The produced aerosol consists of the nanoparticle mixture described by Senden et al. which was found incorporating other isotopes [11].

#### 2.3.3. Process for the Production of a Radioactive Aerosol—US 7722856 or Improved Process for the Production of a Radioactive Aerosol—AU 2006200755

These patents disclose an improved method for Technegas production that overcomes the following three disadvantages associated with the previous methodology: (1) remove contamination from the soluble free radionuclide due to a rapid sublimation of sodium chloride; (2) remove insufficient resistive heating of the carbon crucible that leads to generation of a non-optimum amount of the imaging product; and (3) identify the length of heating and residency time prior to heating that were not disclosed in Patent No. 5064634. In combination, those patents describing a method for operation that involves sublimation in an argon atmosphere in a chamber purged considerably within 2 to 10 min. The sublimation temperature was considered to range between 1200 and 1800 °C and to last for a duration between 10 and 20 s. Subsequently, the ablation temperature is between 2740 and 2780 °C with a rise time between 0.3 and 0.7 s. This results in efficient production of the sublimation of the salt and ablation and formation of the Technegas particles in less than 3.5 s [12,13].

It is interesting to note that there are other patents filed with inventions related to Technegas, in which the technology has been used in applications for other clinical purposes. These include a Method for Detection of Fibrin Clots—US 6977068. This patent resulted in the technology ThromboTrace^®^: a product is formed by electrostatically dispersing Technegas particles in injectable aqueous solution (such as 5% glucose) using a Precipitator technology—US 5792241 [14]. The intended use of this product is to be injected intravenously as a radiotracer for locating the developing deep vein thrombosis in humans, with the invention being proven in preclinical and Phase 1 studies [15].

## 3. Physicochemical Properties of Aerosol

### 3.1. Aerosol Particle Size and Geometry

The aerosol particle size of Technegas has had a wide range of reported values, using numerous techniques over the years, with size ranging from a few nanometres to hundreds of nanometres. This variation in reported size (and composition) has caused some controversy within the scientific community and many follow-up studies. Subsequently, there is an extensive understanding of the physicochemical aspects of this material as well as a detailed understanding of particle size methodology. Here, we report historical findings and discussion relating to particle size, characterisation, and methodology as well as highlight recent studies that overcome some of the earlier approaches and challenges relating to Technegas measurement. 

In 1986, Burch et al. examined Technegas with electron microscopy and electron energy loss analysis. They described Technegas as radioactive ‘soot’ due to its appearance and reported it to be structured aggregates of carbon at a size ≤ 5.0 nm in diameter [16]. Three years later, Strong and Agnew estimated the aerosol particle size to be above 100 nm. Strong and Agnew studied the particle size of Technegas using a multichannel diffusion battery, which sampled the aerosol through several parallel screens with collection filters. Filters were collected and analysed for radioactivity and a particle size distribution generated [17]. Strong and Agnew reported a mean particle size of 140 nm with a GSD of 1.5, over a 4.5 min sampling period. Additionally, the team reported that there was little difference in the particle size distribution with respect to sampling time when measurements were taken at 1, 4.5, and 8.5 min. Using these data, Strong and Agnew described the fractional deposition in the tracheobronchial (TB) and parenchymal (P) regions for aerosols with aerodynamic median diameters of less than 500 nm. The study proposed that particles with a diameter of 140 nm were estimated to have a fractional deposition of 0.05 in the TB and 0.2 in the P region. Considering Technegas particle size, a higher fraction of Technegas aerosols would deposit in the P region for good-quality gamma-camera images [17]. 

**Figure 6:**
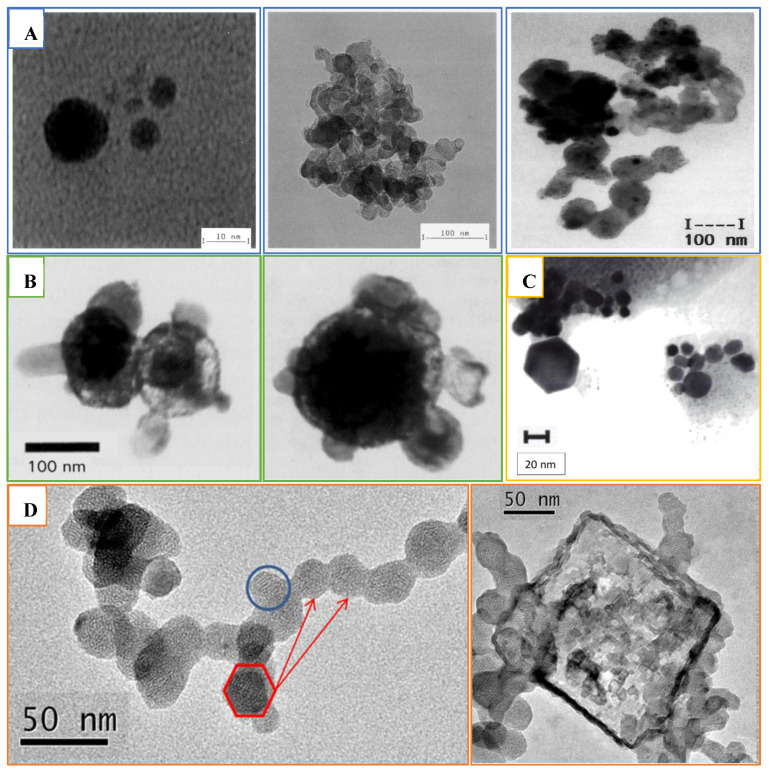
Technegas particle transmission electron microscopy images as obtained through the years using different techniques or methods. (**A**) Primary particles (left), agglomerate (middle), and particles in the gas stream (right) [18,19]; (**B**) particles in the gas stream (left and right) [20]; (**C**) cluster of particles precipitated in liquid. Reprinted/adapted from [7]; (**D**) primary hexagonal particles agglomerated in clusters (left, red hexagon and red arrows) and NaCl crystals in aerosolized nanoparticles (right) [21]. Reprinted/adapted with permission from Refs. [18,19,20,21]; 1993, 1994, 1995, 2021, Springer Nature.

In the 1990s, Lemb et al. [18] evaluated the Technegas particle’s structure and measured its size after passing the gas through two solvents: isopropanol and water. Collected samples were analysed using transmission electron microscopy (TEM), photon correlation spectroscopy (PCS), and time-of-flight mass spectroscopy (TOF-MS). The obtained data revealed Technegas consisting of carbon or graphite particle agglomerates (Figure 6B) with a size range between 60 and 160 nm. These larger agglomerates were composed of primary particles in a size range between 7 and 23 nm (Figure 6A). Compared to the original report by Burch et al., Lemb and his colleagues did not detect particles in the 1 nm range. Lemb et al. reported the particles to be hydrophobic and soluble only in organic solvents. Furthermore, in line with Strong and Agnew’s conclusions, Technegas was concluded to be superior to other conventional aerosols used for lung ventilation studies in nuclear medicine. This conclusion was supported by the fact that Technegas particles were of a size around 100 nm with a hydrophobic nature which enhanced size stability in a humid lung atmosphere [18].

A year following the Lemb et al. publication, Burch et al. questioned the correctness of the Lemb et al. work in a letter to the editor of the *European Journal of Nuclear Medicine* [19]. He explained that Lemb et al.’s findings were suspicious due to inaccurate interpretation of the experiment as well as unsuitable sampling procedures. Burch et al. examined Technegas particles using electron microscopy from the gas stream, while Lemb et al. scanned the extracted particles of Technegas after collection from liquid using TEM. It was proposed that the complicated nature of Technegas particles created pitfalls when studying the physical behaviour of small particles in the nm size range. For instance, Technegas particles would aggregate into larger agglomerates or clusters whenever the particles were brought into close contact to each other. 

Lemb et al. rebutted Burch et al.’s claims with support using unpublished results and their historical experience with using Technegas [19]. They collected TcC particles from a Technegas stream on a TEM grid and examined them on TEM. An average of 20 particles were detected in the TEM images (Figure 6C), and they were all of a size and structure matching those particles observed by TEM from liquid collection in their previous publication. The collected particles directly from Technegas stream were reported as more representative to what the patient inhales. In accordance with the observed morphology of these aggregates and from observations of other materials evaporated in gas atmospheres, it could be concluded that aggregation occurred in the hot gas zone of produced TcC and not as a result of particle assembly after collection in liquid as proposed by Burch et al.

In 1995, Lloyd et al. studied Technegas and Pertechnegas to investigate factors that may impact Technegas particle size distribution in standard clinical use [20]. Technegas is produced in an inert argon environment, while Pertechnegas requires an oxygen/argon mixture to produce technetium oxides. As with the Strong and Agnew study, Llyod et al. used a diffusion battery for measurement of the aerosol particle size; however, they included a primary impactor stage to remove 500 nm particulates. The activity size distribution of both Technegas and Pertechnegas was similar and fitted a log-normal distribution. The activity median diameter and GSD were 158 nm and 1.5 for Technegas, while 167 nm and 1.6 was recorded for Pertechnegas. Consequently, the difference in their physiological behaviour could be related to differences in their chemical nature or composition and not attributed to their physical properties. Hence, it was likely that both gases would have similar distributions after lung deposition. 

In contrast to Burch et al., Lloyd et al. reported that only 1% of the total radioactivity was within particles of the size ≤ 5 nm. Furthermore, in general, the particle size was similar to that reported by Lemb et al. and, for aggregates, the findings of Strong and Agnew. Interestingly, aerosol aging was reported at 8.5 min with an observed increase in activity median diameter from 158 to 225 nm. This difference was attributed to the method setup and the inclusion of an impactor stage in the Lloyd et al. study. Additionally, it was reported that particle size increased in response to the number of simmers used, with activity median diameters of 158, 167, and 194 nm being reported for 1, 2, and 3 simmers, respectively. This was explained to be due to an increase in salt content over multiple simmers and was confirmed by conducting a low salt vehicle study in which the activity median diameter was 102 nm. Furthermore, TEM analysis (Figure 6D,E) of particles collected onto copper grids directly from a stream of Technegas showed the presence of agglomerations with total dimensions between 100 and 300 nm, correlating well with previous findings.

A geometrical description of Technegas particles was reported in 1997 by Senden et al. by sampling the Technegas particles using electrostatic precipitation [22]. The TEM images showed metallic technetium encapsulated within a layered graphite or carbon matrix in near-perfect hexagonally shaped platelets (Figure 6F). The average particle size of 80% of the particles was below 100 nm, with a particle width of around 30–60 nm and thickness around 5 nm. Most of these platelets had a thickness to diameter ratio of about 1:10. A possible controlling factor for changes in particle size is the rate of cooling (Figure 5). It was proposed that during the vapor condensation step a drop in temperature of only 300 °C would limit the collisions within the vapour phase, limiting particle growth [7].

More recently, Blanc-Béguin et al. compared the physical properties of carbon nanoparticles labelled with either ^68^Ga or ^99m^Tc prepared using a Technegas generator following the standard clinical procedure [21]. Collection was achieved using an airborne aerosol sampler with collection on filters or TEM grids. TEM images revealed the presence of large secondary clusters or agglomerated hexagonally structured primary particles (Figure 6G). Both showed a layered structure, implying that gallium or technetium were covered with a layer of graphite positioned in parallel to their surfaces. The primary particle size was close for both types of aerosols, with a primary mean diameter of 20.9 ± 7.2 nm and 19.8 ± 11.7 nm for ^99m^Tc-labelled and ^68^Zn-labelled (decayed Ga) carbon nanoparticles, respectively. These data are comparable to the previously reported particle size of ^99^Tc-labelled carbon nanoparticles, which suggests ultrafine particles for both nanoparticles. In this study, NaCl crystals (Figure 6H) were also observed in the TEM images of carbon nanoparticles in the shape of quadratic crystals [21].

### 3.2. Limitations of Particle Size Distribution Analysis and Application of Cascade Impaction

As discussed previously, numerous methods have been used to assess the particle size and size distribution of Technegas, with each having advantages and disadvantages. The three common approaches are (1) collection in liquid by passing the air stream through a suitable chamber, (2) collection from the chamber via sedimentation, and (3) collection from the generator output by sampling the air stream directly. The latter method can be subclassified into (a) ‘whole’ air sampling (i.e., collection of a representative sample on a filter or sample grid) and (b) size classification apparatuses that separate out particle size distributions through filtration. 

Examples of liquid extraction processes include the work by Lemb et al. in which Technegas particles were collected by passing the output stream through aqueous or nonaqueous solvents before subsampling [18]. Examples of collection via sedimentation include the work by Jackson et al., who collected samples on a stainless-steel probe or copper SEM grid mounted inside the main chamber [23]. Similarly, Isawa et al. placed holey carbon films (or colloidion films) directly into the chamber and collected after sedimentation for 1 day [24,25]. In terms of whole air sampling, Blanc-Béguin F. et al. collected aerosol directly from the exit inhalation tubing using an aerosol collector. 

As all the above methods collect the whole aerosol on solid substrates (with the exemption of the liquid capture method), subsequent particle size distributions must then generally be evaluated using microscopy and image analysis techniques. This generates issues with ensuring that sampling is representative, since the number of particles evaluated will be only a tiny fraction of that produced. Furthermore, collection in liquids (or resuspension of filtered samples in liquids) can be subsampled for laser scattering methods; however, care needs to be taken to ensure that aggregation or segregation does not occur due to Van der Waals, DLVO, and surface tension forces that would give a sample bias different from the Technegas properties in air.

**Figure 7:**
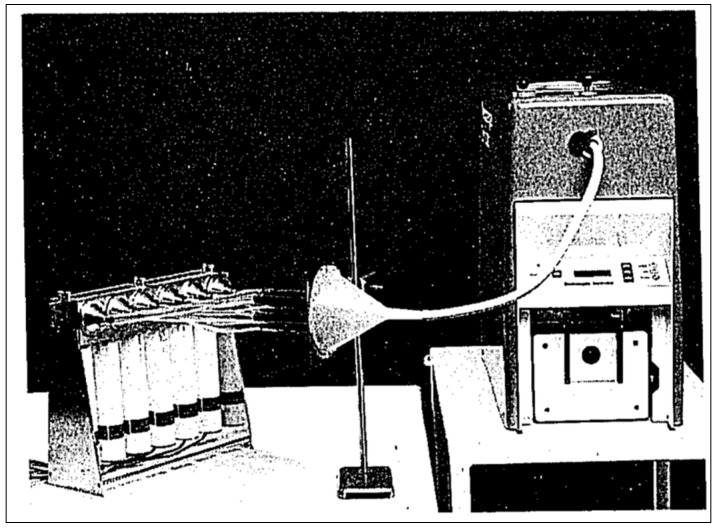
Photograph of a parallel diffusion battery connected to a Technegas generator. Reprinted/adapted with permission from Ref. [17]. 1989, Wolters Kluwer Health, Inc.

Size classification through filtration overcomes the aforementioned issues as it samples the whole aerosol and classifies it through filtration or impaction. Lloyd et al. is a good example of the filtration approach, where they reported the particle size of Technegas using a diffusion battery [20]. The diffusion battery works on the principle of filtration (tortuosity) of the particles as they pass through filters with different particle size capture efficiency. In the case of Lemb et al., these covered a range of 5–500 nm with a final wool filter stage and primary impaction stage to remove everything greater than 500. Particle size in this system can be determined by measuring the radioactivity on each screen. Lloyd et al. used a serial diffusion setup; however, this can also be assembled in a parallel configuration as in the earlier work by Strong and Agnew [17] (Figure 7). 

As with all methods, the diffusion battery also has limitations. First, the equipment will only produce radioactive particle size since the resulting particles are generally analysed using a gamma counter. Second, particles are measured in terms of filter porosity and not aerodynamic diameter (the latter being more useful when assessing respiratory deposition). Third, such techniques make it difficult to conduct subsequent image or chemical analysis since collection from the screens has analytical challenges.

One approach to overcome the sampling issues encountered in the above methods is to use cascade impaction. Cascade impaction is an industry standard for testing therapeutic aerosols, and the *United States Pharmacopeia* Chapter <601> establishes methods, validation requirements, and apparatuses that can be used for routine quality control. Cascade impactors work by inertial impaction and classify an aerosol onto a series of stacked plates by passing the air stream through a series of jets with decreasing size. Pharmaceutical impactors are not suitable for evaluating Technegas, since they are designed for conventional nebulisers, metered dose, and dry powder inhalers that have much higher particle sizes (ca. 1000–6000 nm). Low-pressure impactors work on the same principle, and there are commercial systems designed for evaluating submicron particulates, including environmental pollution and carbonous exhaust particulates for the automotive industry. The electrical low-pressure impactor (ELPI) is one such device. 

#### 3.2.1. Application of the Electrical Low-Pressure Impactor to Evaluate Technegas Particle Size

The electrical low-pressure impactor allows measurement of real-time particle size distribution and concentration across a wide size range suitable for evaluating Technegas. The current ELPI (ELPI+, DEKATI, Finland) has a working range of 6–10,000 nm and collects data at a rate of 10,000 measurements per second.

**Figure 8:**
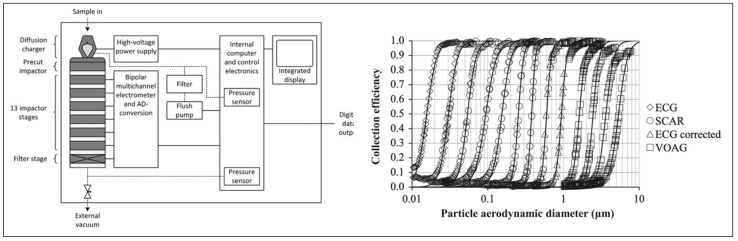
A schematic of the operating principles of the ELPI and particle size distributions on each stage from calibration data. Reprinted/adapted with permission from Ref. [26]. 2014, Elsevier.

In simple terms, the equipment works by charging an incoming aerosol and then measures the electrostatic charge as particles deposit on each impaction stage. With a knowledge of the cut-off diameter of a given stage and the charge measured, it becomes possible to report the aerodynamic size distribution in real time. Furthermore, particles deposited on each stage may be collected for radioactive or chemical quantitation, allowing the measurement of aerodynamic radioactive or chemical distributions as well as being available for collection for microscopic analysis. A schematic of the operating principles of the ELPI and particle size distributions on each stage from calibration data is shown in Figure 8 [26].

**Figure 9:**
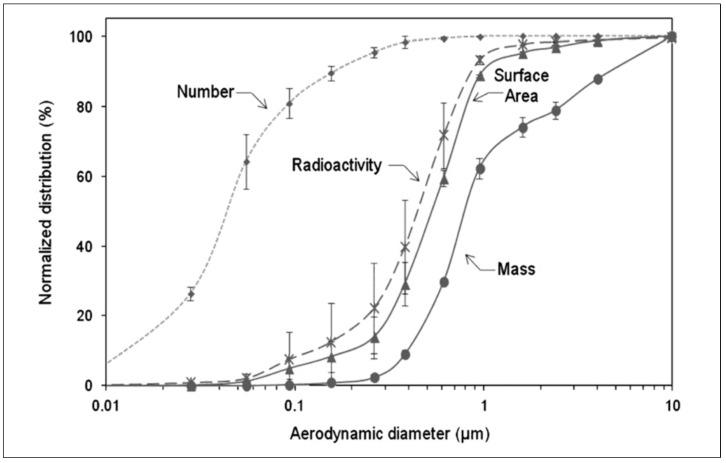
Aerodynamic size distributions (cumulative) of Technegas aerosol using an ELPI and gamma counter. Reprinted/adapted with permission from Ref. [27]. 2013, Elsevier.

Pourchez et al. utilised the ELPI in combination with a gamma camera to determine the radioactivity aerodynamic diameter as well as the particle size distribution by weight, number, surface area, and radioactivity of Technegas [27]. With the capability to measure both the aerodynamic mass and aerodynamic radioactivity, it becomes possible to deconvolute the contributions of different components of the formulation to the inhaled particle size of Technegas. For example, using the conventional clinical settings and sodium pertechnetate saline solution, a plot of mass, radioactivity surface area, and particle number as a function of aerodynamic diameter shows stark differences (Figure 9).

In general, Pourchez et al. found that the mass median aerodynamic diameter (MMAD) (820 nm) of Technegas was significantly higher (20×) than the activity aerodynamic diameter (AMAD) (450 nm). This finding corresponded to half of the overall mass and could be attributed to the high number of nanoparticles with significant radioactivity. Furthermore, the higher MMAD when compared to AMAD could be attributed to larger sodium chloride particles generated from the saline component that had negligible radioactivity. In this landmark paper, Pourchez et al. additionally studied the impact of simmer time, burn time, burn temperature, and residency time using this technique [27].

#### 3.2.2. Chronology of Particle Size Studies, Methodology, and Findings

A summary of Technegas sampling approaches, particle size analysis methodology, and findings over the years is outlined in Table 1. 

It is interesting to note that while the diameter of Technegas has numerous reported values, historical measurements were collected using a multitude of different techniques that yield different size descriptors. These include geometric diameter, volume diameter, aerodynamic diameter, and aerodynamic activity diameter. Furthermore, some of the historical studies investigate the aerosolized Technegas in its agglomerated inhaled form, while others investigate the size of the primary particles. Importantly, throughout all these studies, the particle diameter of Technegas was reported as less than 1 µm, and the activity aerodynamic diameter <500 nm. Given that the analysis of the Technegas particle has repeatedly been demonstrated to be under 1 µm, the ultimate deposition of the radiopharmaceutical in the periphery of the lung at the alveoli level makes it a strong indicator for functional ventilation imaging [28].

To date, the ELPI appears to be the most appropriate technique for sampling Technegas aerosols for particle size measurement. The device can measure particle count, surface area, and aerodynamic mass in real time as well as be used to quantify chemical or radioactive mass distributions on each stage. Furthermore, the ability to collect samples directly from individual impactor stages enables additional opportunities for microscopy and surface analysis.

### 3.3. Composition

**Figure 10:**
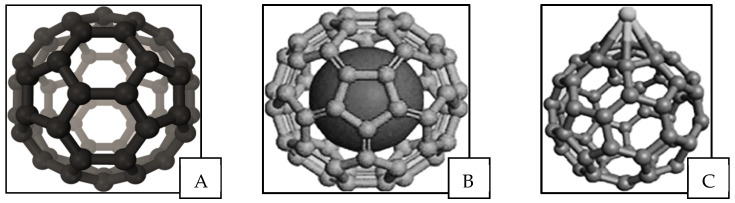
Structure of (**A**) buckminsterfullerene molecule, C60, metallofullerenes, (**B**) endohedral, (**C**) exohedral.

Technegas particle composition and structure have been described using different assumptions and techniques over the years. In the early 1990s, Mackey et al. used a gamma camera to capture momentary dynamic creation of Technegas at the evaporation stage from the graphite crucible [29]. It was observed that Technegas was generated in a pulse-like nature at a temperature of around 2500 °C with a threshold for generation of around 2250 °C. The vaporisation of ^99m^Tc atoms as well as the crystalline graphitic layers of the crucible occur simultaneously, and it was proposed that ^99m^Tc could incorporate into fullerenes. The presence of buckminsterfullerene (Figure 10A) and other fullerenes was confirmed by Mackey et al. after analysing the gas using negative-ion laser desorption Fourier transform mass spectrometry by collecting it as a film on a stainless-steel substrate. It was proposed that fullerene structures formed in the process of generating the Technegas can transform into metallofullerenes due to a technetium atom being attached to the fullerene either in endohedral (Figure 10B) or exohedral form (Figure 10C) [29]. Interestingly, Mackay et al. discussed the previous work of Lemb et al., highlighting that they reported agglomerated particles at 97 nm and primary particles at 10 nm, noting that a C_60_ fullerene has a diameter of 0.7 nm. This is interesting since it does not address whether the primary particles reported by Lemb et al. contain fullerenes or that fullerenes are present but undetectable using conventional size techniques. In addition, Mackey et al. reported that the purity of argon gas used had an observable impact on the nature of the produced gas. For instance, the use of argon gas with a 3% oxygen during the gas generation process led to the production of a chemically different ventilation agent, Pertechnegas. In the Pertechnegas, no fullerene ions were found with mass spectrometry showing a range of technetium metal oxides [29]. 

Lloyd et al. suggested that the mechanism of formation could not be purely the aggregation of fullerenes since the final particle size was also dependent on salt concentration [20]. They proposed the formation of a vapourised salt aerosol during the temperature ramp followed by condensation of carbon around these molecules to form nucleation centres for generation of Technegas. Therefore, a high salt content would produce a high number of primary particles, which leads to a higher coagulation rate and larger agglomerated particles. Lloyd et al. went on to use this theory to explain why Technegas differs clinically from Pertechnegas. When oxygen is present, the interaction with carbon vapor hinders coagulating of carbon around the salt nuclei. This allows the formation of a soluble ^99m^Tc-labelled salt aerosol lacking a protective outer carbon layer. This protective outer carbon layer in Technegas gives it a hydrophobic property which leads to slower clearance when imaging [20]. 

Isawa et al. reported the structures of Technegas by collecting sedimented particles from a generator [24]. Importantly, they reported hexagonal structures for technetium metal with two distinct types of particles. On rare occasions, particles that resembled carbon nanocapsules were observed; however, more often, crystals with a boundary halo consisting of amorphous carbon was seen. Interestingly, Isawa et al. reported that carbon films present on Technegas were not present on Pertechnegas, indicating that while particle size remained similar, the presence of carbon films drove the difference in clinical properties as highlighted by Lloyd. Isawa et al. also evaluated particle size by SEM. In their analysis, the team reported two size distributions of ~5 nm and larger spherical particles of ~50 nm. The larger particles were reported as being NaCl; however, there was no indication how this was identified or why these had not crystalised into a standard cubic structure [24]. 

Llyod et al. questioned both the size analysis and methodology underpinning this study [25] since particle sizing was conducted on limited particles after sedimentation and measured in terms of physical diameter rather than activity diameter (which would consider volume). Interestingly, Isawa rebutted Lloyd et al.’s letter to the editor but also stated that samples were left overnight before sampling [25], potentially impacting the properties of the resulting particles (particularly the hydroscopic salt). 

During the same period, Jackson et al. used scintigraphic methods including X-ray photoelectron spectroscopy (XPS) and scanning transmission electron microscopy (STEM) coupled energy dispersion X-ray analysis (EDS) to study the chemisorption of pertechnetate on graphite [23]. In general, they reported Technegas to be either (TcO_2_)n or (TcO_2_)n bound to carbon nanoparticles exohedrally in a fullerene structure. In addition, the chemical form of technetium in the crucible residue was found to differ from that detected in the aerosol particles. Jackson et al. reported the size of ^99m^Tc-containing particles to be 10 and 80 nm, which agreed with the results of Lemb et al. and others; however, EDS from Technegas particle measurements varied from those obtained by other researchers in terms of carbon signal related to the carbon particles’ encapsulation of metals. 

Senden et al. [7] proposed the native metal was the main composition of the primary Technegas particles with no presence of oxides or hydroxides. This finding confirmed Fawdry and Gruenewald’s assumptions of the impossibility of Technegas aerosol to contain technetium oxides due to the high temperature used in the generation process. Therefore, volatile species (e.g., Tc_2_O_7_, TcO_2_, and NaTcO_4_) would be eliminated, and technetium halides are unlikely to form. They suggested elemental technetium and technetium carbide as the main forms of technetium under the furnace production conditions [9]. 

During analysis, Senden et al. reported near-perfect hexagonal platelets, which would be consistent with the hexagonal closed lattice for technetium. Furthermore, the team found no evidence of a body-cantered cubic phase for TcC in the resulting aerosol and, instead, a layer of partially ordered graphite coating the surface of the technetium platelets, effectively encapsulating the metal crystal in carbon. Additional studies of crucible residues revealed the crucible interior had technetium-rich particles of 500 nm, composed only of technetium and carbon. The overall structure of these residues was technetium-rich spheres enveloped by equal quantities of carbon, technetium metal, and TcC. Senden et al. concluded that salt played no role in the growth of the primary Technegas particles due to no significant activity observed at the NaCl evaporation temperatures. Furthermore, they concluded that the lack of TcC in the resulting aerosol may potentially be due to decomposition. Lastly, the Senden et al. study discussed the fullerene model proposed by Mackey. They concluded that the vast majority of Technegas aerosol could be accounted for as hexagonal platelets, meaning that for a fullerene species to be the active species, it would have to exist in populations around a million times the number of observed platelets. Furthermore, the conditions for fullerene production were far from optimal in such a generator; in fact, it could be proposed that the carbon film is actually a mesoscopic fullerene structure present on the platelet surface [7]. 

Jackson et al. in 1998 commented on Senden et al.’s work, drawing from several literature sources to suggest that the carbon-coated crystals were not the only species present within Technegas. For instance, Jackson highlighted the Isawa publication in which two species were reported. Additionally, Jackson et al. drew on other literature that studied mid-transition metals and the formation of small nanometre crystallites that acted as templates for carbon nucleation and growth while larger crystals were coated in the manner suggested by Senden et al. [30]. In response to Jackson et al., Senden et al. reasserted their proposal that the main radiotracer species in Technegas is crystalline technetium metal in a nanometric-size range encapsulated by graphitic carbon layers. Senden et al. indicated that many of the cited works in the Jackson et al. letter to the editor studied carbon encapsulation through completely different methodologies and conditions to that in a Technegas generator (in some cases using temperatures surpassing 4000 °C, introducing ionization processes). Senden et al. also asserted that the suggested link between particle size and encapsulation was invalid since the encapsulation process would be independent of size and would be a simple matter of surface thermodynamics. In simple terms, they proposed that the high surface energy metal condensate would be lowered by the presence of carbon molecule attachment. Since the metal cannot react with the carbon, the two phases would stay separate, and the carbon would form a skin around the metal crystallite. [30]. 

In a trial that estimated the radioactivity of the primary Technegas particles, Blanc-Beguin et al. reported that a percentage of more than 80.5% recovered radioactivity was attributed to particles with a size of < 80 nm. Therefore, it was concluded that the generated primary carbon nanoparticles were composed of radioactive metal with a diameter of less than 80 nm [21].

### 3.4. Pertechnegas and Technegas

As previously discussed, Technegas forms in an inert argon environment, while Pertechnegas forms in the presence of oxygen (in an argon/oxygen mix). The differences between Technegas and Pertechnegas in terms of formation conditions and composition were studied by Scalzetti and Gagne in 1995 using thin-layer chromatography (TLC) [31]. The study assessed the behaviour changes of the generated gas as a function of the level of oxygen in an argon–oxygen mixture and monitored the transition between Technegas, Pertechnegas, and other related radioaerosols at different oxygen concentrations. An abrupt transition from Technegas into Pertechnegas was observed between 0.1% and 0.2% levels of oxygen. In the presence of pure argon, ^99m^Tc pertechnetate (^99m^TcO_4_^-^) heated inside the graphite crucible undergoes reduction. Therefore, the formed mixture contains insoluble metallic technetium and ^99m^Tc carbides along with soluble ^99m^TcO_2_ formed by incomplete reduction of ^99m^TcO_4_^−^ and reverts to that form in an aqueous media. In the presence of small amounts of oxygen in the argon mixture, the vaporized graphite at high temperatures will combine with the oxygen forming carbon monoxide (Co) and carbon dioxide (CO_2_). The presence of extreme amounts of oxygen constrains the reduction of ^99m^TcO_4_^−^ into ^99m^Tc carbides and metallic technetium and favours the production of ^99m^TcO_2_ and ^99m^TcO_4_^−^. Hence, the purity of argon that is suitable to generate Technegas preferably should be above 99.9% [31].

Another factor affecting the Technegas/Pertechnegas ratio is the operating temperature of the Technegas generator. Burch and Browitt praised Scalzetti and Gagne’s publication that examined the gas phase reaction that accompanies the formation of Technegas and Pertechnegas but additionally highlighted the importance of operating temperature on Technegas that naturally forms by coating the technetium with graphite into micro-aerosol. They found in one set of measurements that the yield of Pertechnegas reduced between 65% and 3% if the operating temperatures were increased from 2475 °C to 2550 °C. Presumably, this is due to an increase in carbon species coating the technetium [32]. 

## 4. Clinical Application

The primary clinical indication for Technegas in nuclear medicine is the diagnosis of pulmonary embolism. Furthermore, Technegas ventilation imaging can be applied in assessing conditions such as chronic thromboembolic pulmonary hypertension, asthma, chronic obstructive pulmonary disease, emphysema, and quantitation before lung volume reduction surgery [4].

**Figure 11:**
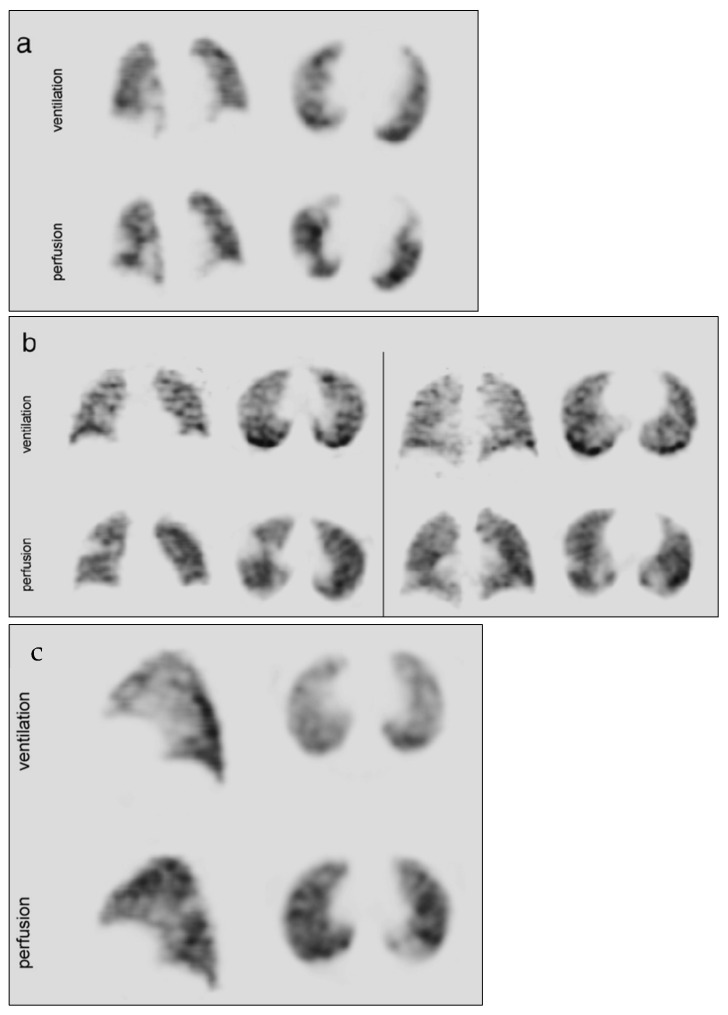
(**a**) A 63-year-old male with a 10-h history of right-sided pleuritic chest pain. V/Q SPECT showed multiple asymmetric wedge-shaped perfusion defects in reference to PE. (**b**) A 37-year-old male who presented with a swollen leg and chest pain. SPECT showed multiple asymmetric wedge-shaped perfusion defects in reference to PE. (**c**) A 31-year-old female who presented with shortness of breath, pleuritic chest pain, and two episodes of haemoptysis. V/Q SPECT showed a single subsegmental asymmetric perfusion defect in the left lung. Computed tomography pulmonary angiography performed the next day showed no PE, but lingular atelectasis, pericardial thickening, and a small pericardial effusion. Reprinted/adapted with permission from Ref. [35]. 2014, Elsevier.

Clinical studies have shown over the years the utility and superiority of Technegas for different diagnostic imaging procedures, especially for lung ventilation/perfusion (V/Q) scanning (Figure 11). For instance, a study was performed on 584 patients between October 2004 and July 2005 for ruling out pulmonary thromboembolism. The study used a series of single photon emission computed tomography (SPECT) pulmonary scintigraphy with ^99m^Tc-Technegas. The main finding was that the obtained images could be used safely for ruling out pulmonary thromboembolism with only a few imprecise studies [33]. In 2002, Suga K described advances in technical and analytical methods in pulmonary ventilation SPECT with technetium-99m (^99m^Tc)-labelled Technegas. First, respiratory-gated image acquisition of ^99m^Tc-labelled Technegas SPECT eliminated challenging effects of the SPECT images acquired during non-breath-hold. Second, a fusion image between Technegas SPECT and chest CT images produced by a fully automated image registration algorithm provided complementary information on function and anatomy. Third, new analytical approaches by means of fractal analysis, or the coefficient of variations of the pixel counts for Technegas SPECT data, assisted the objective evaluation of ventilation abnormality levels [34]. 

**Figure 12:**
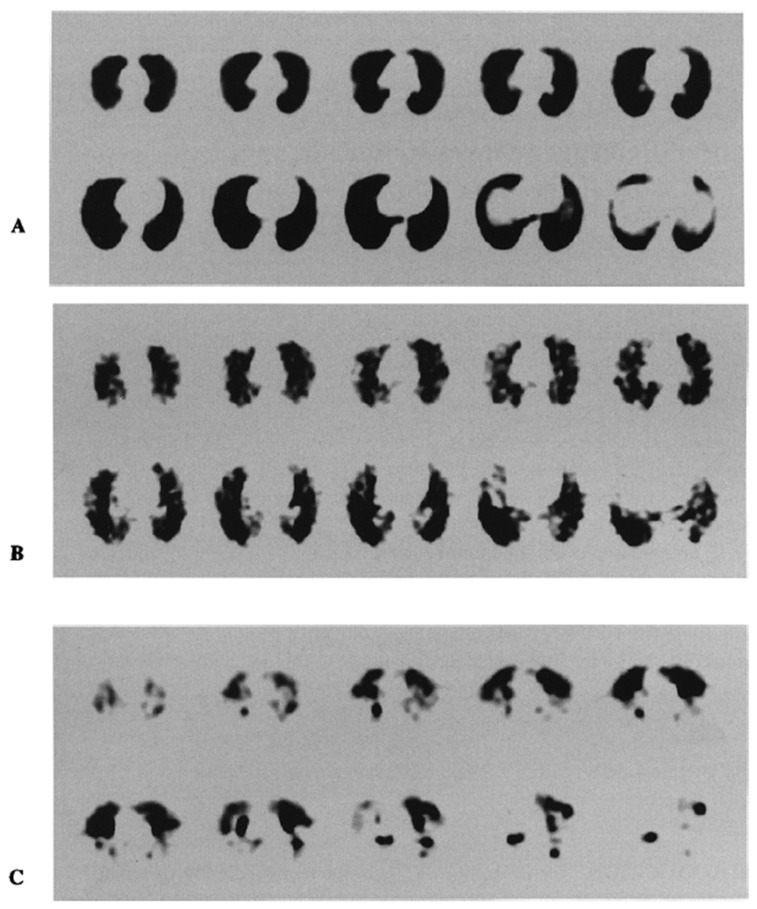
(**A**) A 45-year-old healthy male. Technegas SPECT images show homogeneous distribution in whole lung. (**B**) A 65-year-old male with mild emphysema. Technegas SPECT images show heterogeneous distribution in whole lung. (**C**) A 63-year-old male with severe emphysema. Technegas SPECT images show heterogeneous distribution of cold and hot spots throughout peripheral lung field. Reprinted/adapted with permission from Ref. [36]. 2002, Springer Nature.

For emphysema diagnosis and severity, Nagao and Murase demonstrated the capability of Technegas SPECT images to reveal peripheral irregularity in mild emphysema, hot-spot growth, and regional defects in severe emphysema (Figure 12). In addition to the clearly defined heterogeneous distribution of Technegas in images, the study allowed the generation of a diagnostic index for emphysematous severity. This was achievable when Technegas SPECT images were accompanied by 3D fractal analysis (3D-FA) for quantifying the heterogeneous distribution of the gas. After reviewing the results of 25 patients with pulmonary emphysema, the fractal dimension derived from 3D-FA was found to strongly correlate with pulmonary function in patients with emphysema [36]. A study on a 5-year-old girl using combined ^99m^Tc Technegas inhalation with ^133^Xe gas ventilation-^99m^Tc MAA perfusion indicated a probable use for assessing collateral ventilation. Both SPECT images with ^99m^Tc Technegas and ^99m^Tc MAA showed a focal defect in the affected lung, with a smaller defect on the ^99m^Tc Technegas study than on the ^99m^Tc MAA image [37].

For lung cancer, a computer-aided respiratory-gating technique for ^99m^Tc Technegas/MAA SPECT images was used for assessing the functional impairment of regional lung segments in patients with lung cancer. A study involving 23 patients with lung cancer revealed that the technique can provide an accurate and detailed correlation between morphology and function in regional areas of the lungs. Thus, a comprehensive evaluation of the tumour expansion to the bronchi, pulmonary vessels, and the other lung lesions was achievable. In addition, the obtained images enhanced the prediction accuracy of the respiratory function after operation by ensuring a precise positioning for the lung lobes of interest for resection [38]. In another study, an abnormal tracheal deposition of Technegas was observed in the V/Q scanning of a 63-year-old female with possible pulmonary embolism symptoms. The deposition of Technegas was seen in the carina region on the ventilation scan, and a CT scan revealed an endobronchial mass relevant to the deviation on the ventilation scan. A suspicion of endobronchial malignancy was confirmed by biopsy and found to be non-Hodgkin lymphoma. The deposition of Technegas on the mass projecting to the tracheal lumen caused the observed V/Q scanning deviation, which was useful in revealing a possible malignancy that required investigation [39]. 

In the case of Graves’ disease, V/Q scintigraphy showed abnormal concentrated and uniform accumulation of radioactive material in both thyroid gland lobes in a patient. Although activity is rarely seen extrapulmonary in V/Q scintigraphy, its existence usually indicates the presence of underlying conditions. Thyroid uptake of Technegas, a ventilation tracer, may indicate hyperthyroid states, such as in Graves’ disease [40]. 

Lastly, although Technegas for clinical use in lung imaging is controlled via radiopharmaceutical regulation, the US Nuclear Regulatory Commission subcommittee did not forbid its use in nursing mothers. However, they recommended that breastfeeding be stopped for 24 h after administration of Technegas. Similarly, the International Commission on Radiological Protection suggested that nursing should be disrupted after the administration of Technegas. Experts recommended, for the purpose of reducing radiation exposure to the infant, that the infant should be breastfed prior to Technegas administration and then not be fed for 3–6 h, followed by expressing and discarding the whole milk one time. While this is not a direct clinical application, it does indicate the safety of Technegas use in complicated patients groups such as nursing mothers [41]. 

## 5. Comparison with Other Devices

There are limited sources of radioactive gases suitable for ventilation imaging. In addition, some of the available radionuclides, such as xenon-133 and krypton-81 m, are compromised by many general factors such as the availability, gamma energy, available radiation dose, and cost. Furthermore, the noncolloidal nature of such a gas limits potential applications outside of perfusion studies. 

**Figure 13:**
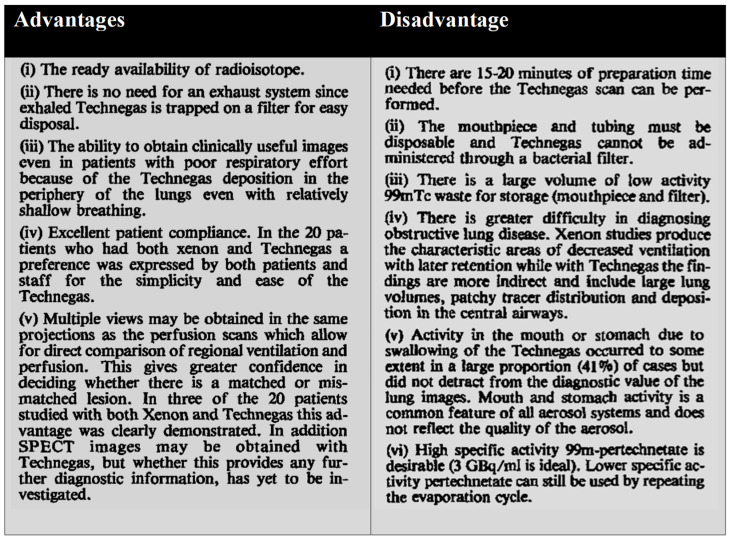
Advantages and disadvantages of Technegas compared to xenon scans. Reprinted/adapted with permission from Ref. [9]. 1988, John Wiley and Sons.

In comparison, aerosols of technetium-99m compounds were designed to overcome the limitations of radioactive gases. In a study involving 196 patients, high-quality Technegas images were obtained within 20 min procedure time, mostly after one inhalation, which aided in patient convenience. Technegas deposits in small airways and the alveolar region, while xenon-133 remains in equilibrium during scanning. Therefore, a significant volume of conduction airways signalled in the inner zone from xenon-133 transiting in and out of the lung [16]. In another clinical study of 150 patients, it was reported to be more comfortable and easier to use Technegas from a patient perspective compared to the xenon spirometer. Advantages and disadvantages of Technegas in comparison to xenon scans are detailed in Figure 13 [9]. Nagao and Murase described Technegas images to be similar or even better at providing diagnostic information for lung ventilation imaging compared with ^133^Xe and ^81m^Kr. Importantly, ^81m^Kr and ^133^Xe scintigraphy requires a ventilation system, while Technegas is available, easy to use, low-cost, and delivers a low dose of radiation [36]. 

The respiratory delivery of nebulized diethylenetriaminepentaacetate (DTPA) labelled with indium-113m or ^99m^Tc requires deep tidal breathing for several minutes. The delivery efficiency is usually low, especially for patients with chronic respiratory disease in which inhalation and exhalation for long periods in closed systems may lead to a low count rate [42]. The lung deposition relies on the aerodynamic particle size of the aerosol ranging between 0.5 and 2 µm for ^99m^Tc-DTPA droplets. Obstructive airway disease causes a nonuniform deposition of ^99m^Tc-DTPA radioaerosol at the central/large airways more than peripheral penetrations. Therefore, the submicron size of Technegas and the need for a few breaths can lead to more efficient deposition, especially in patients with obstructive airway disease [42,43].

In 2010, a head-to-head study thoroughly investigated the differences in ventilation studies performed with ^99m^Tc-DTPA versus Technegas. The study involved 63 patients, of which 35 had obstructive lung disease and 25 without. Both groups were examined with V/Q SPECT using ^99m^Tc-DTPA and Technegas. In both disease groups, the overall irregular distribution of radiotracer and the central deposition level were more marked in ^99m^Tc-DTPA than in Technegas studies. The degree of revert mismatch was less with Technegas because of better peripheral penetration. The degree of focal deposition in distal airways was more pronounced with ^99m^Tc- DTPA in obstructive disease. Mismatched perfusion defects were more commonly found with Technegas in obstructive disease. This intraindividual comparative study illustrates that Technegas is the favoured radioaerosol, mainly in obstructive disease [43].

## 6. Conclusions

The production of Technegas and the Technegas generator has been established for over 30 years and been used in over 60 countries globally for respiratory diagnosis in 4.4 m patients. Scientific understanding of the process of formation and composition of Technegas is well understood, as is evident from the extensive literature available. Scientific instrumentation has advanced over the years, and as these new tools have become available, scientists have rapidly applied them to further our understanding of composition and size. With current technologies such as the electrical low-pressure impactor, it becomes possible, for the first time, to routinely evaluate both aerodynamic activity and mass simultaneously and to analyse the resulting particles with unprecedented surface characterisation tools. While such techniques give us unprecedented insight into the dynamics of Technegas and interpretation of respiratory deposition, the principle that this aerosol is a submicron gas containing agglomerated nanoparticles of carbon-encapsulated technetium has not changed since its invention in 1984.

## Figures and Tables

**Table 1:** Historical review of Technegas particle size and composition investigations using different sampling and analysis techniques.

Year	Sampling Method	Analysis Technique	Particle Size	Morphology/Composition	Reference
1986	Collected from gas stream	EM EELS	Primary particles ≤ 5.0 nm	Radioactive ‘soot’ Aggregates of carbon	[16]
1989	Collected from gas stream	MDB	Median diameter 140 nm and GSD 1.5		[17]
1993	Collected in liquid: water and Isopropanol	TEM PCS TOF-MS	Primary particles 7–23 nm Agglomerates 60–160 nm	Agglomerates of hydrophobic carbon or graphite particles	[18]
	Collected from gas stream	TEM			[19]
1995	Collected from gas stream	Diffusion battery	Median diameter 158 nm and GSD 1.5		[20]
		TEM X-ray microanalysis	Agglomerations of size range 100 to 300 nm	Initial results of composition of sodium and chlorine with a little carbon	
1996	Sedimentation on holey carbon films inside the TP chamber	HR-TEM	Two size distributions: Small particles of ~5 nm Larger spherical particles of ~50 nm	Hexagonal structures of technetium metal with two types of particles: 1- Crystals with a boundary halo consisting of amorphous carbon 2- Particles resemble carbon nanocapsules	[24]
	Sedimentation on a stainless-steel probe or copper grid from the TP chamber	XPS STEM-EDS	Size of particles 10–80 nm	Technegas is either (TcO_2_)n or (TcO_2_)n bound to carbon nanoparticles exohedrally in a fullerene structure	[23]
1997	Electrostatic precipitation in liquid	TEM	Mean size < 100 nm Particle width 30–60 nm Particle thickness 5 nm	Metallic technetium of hexagonally shaped platelets within a layered carbon matrix	[7]
2013	Collected from gas stream by ELPI+	ELPI+ coupled gamma camera	AMAD of 450 nm, GSD of 3.4 MMAD of 820 nm, GSD of 2.7		[27]
		FEG SEM SEM coupled with image analysis	Two particles: Small in 30–300 nm size range Large in the micron size range	Technegas is composed of a predominantly coagulated carbonaceous nature for the ultrafine particles while the dominant NaCl content is in the micron-sized particles	
2021	Airborne aerosol sampler with filters or grids	TEM	Primary particles mean diameter 20.9 ± 7.2 nm	Agglomerated hexagonally structured primary particles covered with a layer of graphite positioned in parallel	[21]

EM, Electron microscopy; EELS, Electron energy-loss spectroscopy; MDB, Multichannel diffusion battery; TEM, Transmission electron microscopy; PCS, Photon correlation spectroscopy; TOF-MS, Time-of-flight mass spectroscopy; XPS, X-ray photoelectron spectroscopy; STEM-EDS, Scanning transmission electron microscopy coupled energy dispersion X-ray analysis; TP, Technegas generator; HR-TEM, High-resolution transmission electron microscopy; ELPI+, Electrical low-pressure impactor plus; FEG SEM, Field emission gun scanning electron microscopy.

## Data Availability

No new data were created in this review article.
